# Books and Pamphlets Received

**Published:** 1886-04-20

**Authors:** 


### BOOKS AND PAMPHLETS RECEIVED.

*A Manual of the Medical Botany of North America* By Lawrence Johnson, A. M , M. D., Lecturer on Medical Botany, Medical Department of the University of the city of New York ; Fellow of the New York Academy of Medicine, and of the New York Academy of Sciences ; Member of the Committee of Revision of the Pharmaeopœa of the United States, etc. New York: William Wood & Co., 56 and 58 LaFayette Place.

A work of nearly 300 large octavo pages, beautifully illustrated. The object of this work is well shown by the following remark in the preface : " As a teacher of medical botany, I have been much embarrassed by the want of a text-book suited to the needs of American students—one combining a brief sketch of general Botany, with descriptions of medicinal plants—and in this volume we have endeavored to supply that want." We regard the work as eminenly instructive and useful.

*A System of Practical Medicine by American Authors.* Edited by William Pepper, M. D., LL. D., Provost and Professor of the Theory and Practice of Medicine and of Clinical Medicine in the University of Pennsylvania; assisted by Louis Starr, M. D., Clinical Professor of Diseases of Children in the Hospital of the University of Pennsylvania. Volumes III and IV.

The first volume of this great work was noticed some time ago, and we have now before os the third and fourth volumes—each gotten out in the same elegant and substantial leather binding, and the one containing 876 large octavo pages, and the other over 1,000 pages. Volume III is devoted to Diseases of the Respiratory, Circulatory and Hæmatopoietic systems ; and Volume IV to diseases of the Genito-Urinary, Muscular and Cutaneous systems, Ophthalmology and Otology. The separate diseases coming under these general divisions are ably and elaborately considered by such writers as Prof. Agnew D. Hays, Prof. Allen Harrison, Prof Da Costa, Prof. S. S. Davis, Prof. Austin Flint, Prof. Beverly Robinson, Prof. B. F. Baer, Prof. Louis Duhring, Prof. William Goodell, Prof. Loomis, Prof. Jacobi, Prof. John Lynch, Prof. A. H. Smith, etc. With such writers, it is expected that the ablest and best is written on any subject submitted to them, and, as a rule, we find this to be true so far as we are capable of judging, upon the parts which we have found time to peruse. Upon the subject of Pneumonia, however, by Professor Loomis, though ably treated in respect to its nature and pathology, we find him opposed to the vast majority of practing physicians in the South in regard to the efficacy of veratrum viride and aconite, etc. He regards these remedies as hazardous, by reason of their depressing action on the heart, and makes the erroneous statement that this class of agents, "and all other cardiac sedatives, which at one time were used so extensively, have now fallen almost entirely into disuse," etc. To this we may certainly say that veratrum viride is still the sheet-anchor in the treatment of pneumonia throughout the Southern States. But our space does not permit a special reference to particular parts of this magnificent work, and we close by recommending it as a great and useful work of reference and for study by the progressive practitioner. Let every one, who can do so, procure the work as the successive volumes appear from the Publishing House of Lea Brothers & Co., Philadelphia.

*Psycheatry*; a Clinical Treatise on Diseases of the Fore-brain, based upon a study of its structure, functions, and nutrition. By Theodore Meynert, M D., Professor of Nervous Diseases, and Chief of the Psychiatric Clinic in Vienna. Translated by B. Sachs, M. D., Instructor in Diseases of the Mind and Nervous system in the New York Polyclinic. Part I—The Anatomy, Physcology and Chemistry of the Brain. New York: G. P. Putnam's Sons. 1885.

This is an able and instructive treatise on mental diseases. The work contains 285 octavo pages, and is illustrated with many cuts showing the anatomy of the brain, etc. Professor Meynert, of Vienna, is noted as a lecturer on diseases of the brain, and for his thorough knowledge of the anatomy and physiology of that organ. The present work is more especially directed to the consideration of the diseases of the fore-brain. His theories and inductions are based upon clinical observation. As chief of the only State Insane Asylum of Austria, he has abundant opportunities for studying the many phases of mental diseases, there being as many as fifteen hundred patients in his clinic. The work is an able one, and well worthy the study of the physiologist, and all students of the brain and mental phenomena.

*Practical Suggestions Respecting the Varieties of Electric Currents and the Uses of Electricity in Medicine;* with hints relating to the selection and care of Electrical Apparatus. By Ambrose L. Ranney, M. D. New York: D. Appleton & Co.

Whether from indifference, scepticism or ignorance it must be admitted that electricity, both as a science and as an art, is disregarded by the great majority of the medical profession. Hence, thus far it has been, in a great measure, appropriated, and dispensed as it were, by a host of quacks, charlatans, mountebanks, street electrifiers, *et id omne genus*. The success which some of these self-asserted Æsculapians have met with, both in their itenerant and dispensary practice, have contributed not only to establish the notoriety of practical proofs but to awaken the profession from their tranquil selfsufficiency and force them to recognize the late achievements of electricity as contributions of almost meridian splendor, to the advancement of medical and philosophical science, and to the relief of our common humanity. In the work of Dr. Ranney the profession cannot fail to find an expose of all modern achievements in electrical therapeutics and in the manifold applications of their wonderful agencies. We commend this valuable guide to the attention of the medical profession, especially to that of the medical student.

The Physicians' Handy Cabinet Battery, as recommended by this author, may be seen illustrated in the advertisement of Wait & Bartlett in another part of this journal.

